# The Louis-Jeantet Prize 2013: Michael Stratton, Peter Hegemann and Georg Nagel

**DOI:** 10.1002/emmm.201202394

**Published:** 2013-01-22

**Authors:** Natascha Bushati, Bernard C Rossier

**Affiliations:** EMBO Molecular Medicine at the European Molecular Biology Organization (EMBO)Heidelberg, Germany; University of Lausanne, and was Secretary of the Scientific Committee

The Louis-Jeantet Foundation annually recognizes innovative biomedical research by awarding a Prize for Medicine to European researchers, who have distinguished themselves in the field of biomedical research. The prize money is meant to be directly invested into ongoing and future research projects of major importance to fundamental or clinical medicine. Since 1986, the foundation has highlighted a remarkably broad range of exceptional research by leading researchers. The inclusion of basic research with biomedical potential in the selection criteria aims to showcase the important role that such research plays in medical innovation.

»Michael Stratton was recognized for the discoveries of the cancer genes Brca2 and Braf…«

The 2013 Louis-Jeantet Prize has been awarded to Michael Stratton (Wellcome Trust Sanger Institute, Cambridge, UK), Peter Hegemann (Humboldt University, Berlin, Germany) and Georg Nagel (University of Würzburg, Germany). Michael Stratton was recognized for the discoveries of the cancer genes Brca2 and Braf and his leading role in the development of the field of cancer genomics. Peter Hegemann and Georg Nagel were acknowledged for the discovery of channelrhodopsin, which founded the field of optogenetics in neuroscience.

As in previous years, EMBO Molecular Medicine is delighted to publish features by the recipients of this year's prize, respectively (Hegemann & Nagel, 2013; Stratton, 2013). In these articles the awardees discuss the genesis and implications of their research from a personal perspective, providing an inspirational read.

After completing a medical degree at Oxford University and Guys Hospital and working as a histopathologist at the Hammersmith and Maudsley Hospitals in London, Stratton switched to molecular oncology research at the Institute of Cancer Research in London. His interest for genome wide analysis led him to the Wellcome Trust Sanger Institute (Cambridge, UK), where he is Deputy Director. His seminal discoveries include the identification of the second breast cancer susceptibility gene, BRCA2, and the implication of the BRAF gene as a major driver of skin cancer, with mutations present in approximately 60% of malignant melanomas. Stratton's discovery and functional characterization of BRAF in melanoma stimulated the development of BRAF inhibitors, which have been proven effective in patients, albeit with the frequent emergence of drug resistance. The BRAF mutations had been discovered as part of the Cancer Genome Project at the Wellcome Trust Sanger Institute, which aimed to map somatic mutations in a wide variety of human cancers. By analysing the exon sequences of 210 diverse primary cancer samples, Stratton and his team further uncovered that somatic mutations in a small number of genes, including BRCA2 or BRAF act as pleiotropic ‘driver’ mutations. In contrast, the majority of mutations mapped are ‘passenger’ mutations in genes that do not significantly contribute to oncogenesis and are infrequently mutated in all cancers. In 2009, Stratton and his team completed sequencing of the first complete cancer genomes from a lung tumour and a melanoma. Many other tumour types have followed as part of another large-scale project, the International Cancer Genome Consortium. Stratton's cancer genome mapping approach continues to highlight possible targets for drug development. His pioneering work has paved the way to modern cancer genomics with direct clinic applications: Specific treatments can be tailored to responsive patients, who carry mutations in specific genes. The long-term vision is that with sinking sequencing costs, cancer genome profiling of patients will become a standard approach for tailored chemotherapy treatment.

»Peter Hegemann and Georg Nagel were acknowledged for the discovery of channelrhodopsin…«

Peter Hegemann and Georg Nagel discovered channelrhodopsins, the light-activated ion channels in unicellular green algae, which control movement of the algae towards or away from light. Peter Hegemann obtained his PhD for studying halorhodopsin at the Max Planck Institute for Biochemistry (Martinsried, Germany). Georg Nagel conducted his PhD work at the Max Planck Institute for Biophysics (Frankfurt, Germany). Hegemann's and Nagel's paths crossed in the early 2000s, when they collaborated on expressing rhodopsin genes from the green alga *Chlamydomonas reinhardtii* into *Xenopus laevis* oocytes. They discovered that these algal rhodopsins, which they termed channelrhodopsins, were a combination of a light receptor and ion channel. Exposure of frog oocytes expressing channel rhodopsin to light opened the channel, making it permeable for cations and inducing electrical currents across the cell membrane. Already back then Hegemann and Nagel realized the enormous potential of channel rhodopsins as tools in biomedical research, opening up the field of optogenetics. Indeed, in the last decade researchers have expressed channelrhodopsins specifically in genetically defined neurons in *Caenorhabditis elegans*, Drosophila, mice and rats. These neurons can then be activated or deactivated by light with certain wavelengths, which allows neuronal circuit mapping and the linking of brain function, behaviour and disease. In mouse models, optogenetics has already showcased the high potential for biomedical applications such as the recovery of vision and optical deep brain stimulation for treatment of Parkinson's disease.

The editors of EMBO Molecular Medicine and the Louis-Jeantet Foundation congratulate the 2013 Louis-Jeantet Prize awardees for their innovative research in molecular medicine.
